# Cancer risk in acromegaly: reassessing the evidence and impact of biochemical control

**DOI:** 10.1210/clinem/dgag230

**Published:** 2026-06-11

**Authors:** Maria Fleseriu, Shlomo Melmed

**Affiliations:** Departments of Medicine and Neurological Surgery, and Pituitary Center, Oregon Health & Science University, Portland, OR 97239, USA; Department of Medicine and Pituitary Center, Cedars-Sinai Health Sciences University, Los Angeles, CA 90048, USA

**Keywords:** acromegaly, cancer, GH/IGF-1 axis, biochemical control, mortality, screening

Although cardiovascular disease, diabetes mellitus, sleep apnea, and skeletal complications are well-documented acromegaly comorbidities (1), association of the disorder with cancer has recently emerged as a contributor to increased morbidity and mortality (1, 2). There is substantial evidence favoring both GH and IGF-1 signaling to promote cellular proliferation, induce DNA damage, suppress apoptosis, and enable a tumor-permissive microenvironment (3). Nevertheless, there has been no compelling epidemiologic substantiation favoring clear-cut cancer risk, a relationship to IGF-1 normalization, or the need for screening regimens.

Prior results of heterogeneously controlled reports from European, United States, and Asian databases have not been consistently uniform. Population-level background cancer rates differ substantially, and reports differ in sensitivity for ascertainment and cancer diagnosis. Furthermore, treatment patterns and surveillance intensity may differ in the countries studied.

Three recent rigorously controlled national cohorts have now been reported that elucidate this important clinical question.

Tsatsaris and colleagues present a well-designed, matched cohort study of 1035 patients from the Swedish Pituitary Register with acromegaly diagnosed between 1991 and 2018, as well as 10 261 population controls. The report substantially advances our understanding, but also raises important clinical questions regarding cancer as a component of the acromegaly phenotype (4). After adjusting for age, sex, and comorbidity, the hazard ratio (HR) for all-cause cancer was increased (HR 1.28 [95% CI, 1.11-1.49]), as was the HR for site-specific colorectal cancer (CRC) (HR 1.84 [1.28-2.64]), and lung (HR 1.95 [1.22-3.11]), hematologic (HR 1.68 [1.03-2.73]), and female breast (HR 1.46 [1.02-2.11]) cancers. Notably, excess cancer risk was already detectable 5 years before acromegaly diagnosis. Cancer-related mortality was elevated only in patients aged 40-60 years (HR 1.48 [1.19-1.85]), while those with persistently elevated IGF-1 levels had higher overall mortality. Counterintuitively, biochemical control did not affect cancer incidence or cancer-related death.

In a nationwide Korean population-based study of 2382 patients with acromegaly and 11 910 matched controls followed from 2006 to 2016 (5), overall cancer occurred in 10.2% of patients vs in 5.9% of controls (HR 1.90 [1.63-2.22]), which is higher than the Swedish estimate (4). The Korean cohort identified the highest risk for brain cancers (HR 6.80 [2.83-16.38]), with increased risks for lymphoma, myeloma, thyroid cancer, pancreatic cancer, and CRC. Similar to the Swedish cohort, cancer risk persisted 5 years beyond acromegaly diagnosis. However, overall cancer risk was disproportionately concentrated in patients aged <50 years, thereby pointing to important age-stratified surveillance implications for acromegaly cohorts.

A study from Israel comprising 470 patients with acromegaly and 2330 control subjects followed for a mean of 10.4 years after diagnosis (7) also showed increased risk of solid malignancies in 21.3% of patients with acromegaly vs 14.8% in controls (odds ratio [OR] 1.6 [1.2-2.0]). Thyroid cancer risk was substantially higher (2.8 vs 0.6%; OR 5.1 [2.3-11.0]), while CRC (3.6 vs 2.8%; OR 1.3 [0.7-2.2]), prostate (2.8 vs 1.7%; OR 1.6 [0.8-3.1]) and renal (1.5 vs 0.8%; OR 1.8 [0.8-4.4]) cancers showed a more modest risk tendency.

Thus, the convergence of these recent independent large nationwide cohorts showing a significantly elevated overall cancer rate in Sweden, Israel, and Korea all appear to suggest that acromegaly could be cancer-risk-modifying.

Whether these observations could be ascribed to pituitary adenomas *per se* was studied in a prospective US cohort of 598 patients with acromegaly and 292 clinically nonfunctioning pituitary adenoma (NFPA) with follow-up extending to 7024 person-years (6). Cancer prevalence by last follow-up was 22.6% in acromegaly vs 12.7% in patients with NFPA (OR 1.99 [1.34-2.97]; *P* = .0005), indicating that excess cancer is likely attributable specifically to GH/IGF-1 excess rather than to the adenoma or its treatment. In contrast to the results of Tsatsaris et al (4), cumulative exposure to IGF-1 excess was a significant cancer predictor in acromegaly (OR 1.278 [1.060-1.541]; *P* = .01), as were age and smoking. This apparently discordant result may reflect differences in the *definition of biochemical control*, which Tsatsaris based on a single IGF-1 measurement not as a cumulative exposure variable. Thus, “current” IGF-1 level might not be a determinant of cancer risk, but chronic exposure to IGF-1 excess over time may be more clinically relevant. This metric underscores the value of cancer prevention by both early diagnosis and sustained biochemical control, even when cross-sectional studies do not fully support this need.

The global ACROSTUDY surveillance of 2077 patients receiving pegvisomant followed for a median of 4.1 years (8) addressed whether IGF-1 normalization on therapy could modify disease-specific mortality risk. Mortality from malignancies was significantly increased with higher on-treatment IGF-1 (HR 1.57 [1.17-2.09]; *P* = .0024), while higher IGF-1 at enrollment (>2× vs <1× upper limit of normal: HR 4.89 [1.09-21.8]; *P* = .038) and presence of malignancy at enrollment (HR 7.05 [2.36-21.03]; *P* = .0005) were the most powerful determinants.

Risk for CRC is most consistently reported in all studies, while other cancer findings suggest unresolved population heterogeneity. Thyroid cancer rates, for example, balance adverse GH/IGF-1–driven signals against surveillance bias (7).

Applying several principles from these reports may lead to improved management. Cancer screening in acromegaly should not solely be contingent on cross-sectionally assessed biochemical disease activity, yet achieving/maintaining IGF-1 normalization is key (1, 2). Colonoscopy remains the cornerstone of CRC surveillance and should be performed at diagnosis. Individualized surveillance intervals incorporating baseline colonoscopy findings, current and historical IGF-1 trajectory, patient age, smoking history, and family cancer history remain to be determined for patients with acromegaly.

Methodologically distinct epidemiologic studies spanning registries, prospective single-center cohorts, and treatment surveillance programs point to a clinically actionable paradigm regarding IGF-1 levels and cancer in acromegaly. The inexorable trajectory and chronic burden of GH/IGF-1 excess across decades, rather than documented single or sporadic IGF-1 elevations, may be the primary pathogenic insult determining the adverse microenvironmental signals that drive cancer excess in acromegaly.

As cardiovascular mortality in acromegaly continues to decline and shift toward neoplastic disease (1, 2), recent evidence identifies IGF-1 normalization as a comorbidity-prevention strategy, and also as a cancer-mortality-prevention intervention. Physicians should translate these results into individualized, evidence-driven care for patients with acromegaly (9).
